# Effects of pension eligibility expansion on men’s memory decline and dementia probability: Findings from the HAALSI cohort in rural South Africa, 2014–2021

**DOI:** 10.1371/journal.pone.0326321

**Published:** 2025-06-25

**Authors:** Janet Jock, Erika T. Beidelman, Meredith Phillips, Lindsay C. Kobayashi, Xiwei Chen, Stephen Tollman, Chodziwadziwa Whiteson Kabudula, Darina T. Bassil, Ryan Wagner, Lisa Berkman, Molly Rosenberg

**Affiliations:** 1 Department of Political Science, Auburn University, Haley Center Auburn, Auburn, Alabama, United States of America; 2 Department of Epidemiology and Biostatistics, Indiana University School of Public Health, Bloomington, Indiana, United States of America; 3 Department of Epidemiology, University of Michigan School of Public Health, Washington Heights, Ann Arbor, Michigan, United States of America; 4 SAMRC/Wits Rural Public Health and Health Transitions Research Unit [Agincourt], School of Public Health, Faculty of Health Sciences, University of the Witwatersrand, Johannesburg, Braamfontein, Johannesburg, South Africa; 5 INDEPTH Network, Accra, East Legon, Accra, Ghana; 6 Umeå Centre for Global Health Research, Division of Epidemiology and Global Health, Department of Public Health and Clinical Medicine, Umeå University, Umeå, Sweden; 7 Harvard Center for Population and Development Studies, Cambridge, Massachusetts, United States of America; University of Oxford, UNITED KINGDOM OF GREAT BRITAIN AND NORTHERN IRELAND

## Abstract

Alzheimer’s disease and related dementias (ADRD) are a growing global health concern, with burdens projected to expand rapidly in the coming decades. Since cognitive decline typically precedes ADRD, it is crucial to identify interventions that may help slow cognitive decline and reduce ADRD risk. We used a quasi-experimental design, exploiting exogenous expansions of South Africa’s Older Persons Grant for men, to estimate its impact on memory decline and ADRD risk in the rural Mpumalanga province of South Africa. We found that expanded pension eligibility was associated with slower memory decline for men who were eligible to receive the pension 5 years earlier [β = 0.027 SD, 95% CI = 0.023, 0.031], as well as for men who were eligible to receive the pension 1−4 years earlier [β = 0.009 SD, 95% CI = 0.004, 0.013]. We also found a 5.2 percentage point lower probability of dementia for men who were eligible for pension 5 years earlier [95% CI = −0.062, −0.032] and a 4.8 percentage point lower probability of dementia for men who became eligible to receive pension 1−4 years earlier [95% CI = −0.062, −0.032]. These findings demonstrate that beyond the policy intent of cash transfers to strengthen individual and household livelihoods, an important further benefit lies in promoting healthy cognitive aging in low- and middle- income countries.

## Introduction

Alzheimer’s disease and related dementias (ADRD) are a significant global public health concern, particularly in low- and middle-income countries (LMICs) where increases in overall life expectancy are likely to result in greater incidence and prevalence of dementia [1–3]. Accelerated memory decline often precedes the development of ADRD [4–7]. Low socioeconomic status (SES) is a consistent predictor of accelerated memory decline and dementia risk [6,8–18]. Thus, interventions targeting socioeconomic determinants in LMICs are crucial for effectively mitigating the rising tide of ADRD cases in the coming decades.

Government cash transfer programs, including pensions, involve direct monetary payments to recipients, thereby providing them with supplemental income. While the key policy intent of pensions in South Africa is to contribute to household income and security, they may also be effective policies for reducing dementia risk. The supplemental income could plausibly impact multiple modifiable risk factors for dementia. Previous studies have shown that cash transfers may support healthy aging by increasing health care utilization, household consumption, improving psychological well-being and child and adult health outcomes [19–34,35]. Moreover, cash transfers may facilitate investment in assets or behaviors that foster social connections and cognitively stimulating activities (e.g., cell phones, transportation), which may help build cognitive reserve and protect against dementia [36,37]. However, globally, there remains a scarcity of evidence employing causal inference to assess the impact of cash transfers in the form of old age pensions on dementia risk. Studies that do exist have shown that pensions tend to be associated with higher cognitive function and slower cognitive decline. A study of the New Rural Pension Scheme income in China found associations between pension receipt and higher cognitive function [38]. Ayyagari & Frisvold [2016b] exploited an exogenous change in the U.S. Social Security income in the 1970s to identify the impact of Social Security income on cognitive function, finding that higher benefits improved the cognitive function of older adults [39,40]. A study in a rural South African setting found that extra income from pension eligibility expansions was associated with higher cognitive function [20]. Three longitudinal studies have found positive associations between cash transfer income and memory function [41, 42, 23]. Studies in South Korea and China found that pension income was associated with slower cognitive decline [41], and slower memory decline [42]. A cluster-randomized trial of social security benefits in Yucatan, Mexico, found that social security benefits were associated with a reduction in memory decline equivalent to five to twelve years of cognitive aging over a six- to nine-month follow-up period [23].

Establishing causal associations between cash transfers and cognitive aging outcomes is difficult with observational study designs due to the endogenous nature of cash transfer receipt. Factors like SES and level of education are likely shared common causes of cash transfer receipt and an individual’s dementia risk. In this study, we exploit exogenous increases in pension income from age eligibility expansions of South Africa’s Older Person’s Grant Pension to estimate the causal effects of increased income on memory decline and dementia risk. The South African government lowered South Africa’s Older Person’s Grant Pension eligibility age for men from 65 years to 60 years between the years 2008 and 2010. As a result, some older men in South Africa were exposed to one to five years of earlier pension eligibility. Our quasi-experimental study design examines the relationship between an exogenous increase in income through earlier pension eligibility and memory decline and dementia. Our prior work in a rural South African study setting found that expanded eligibility for extra pension income was associated with higher cognitive function [20]. This study expands on this prior work by using a longitudinal analysis to assess the impact of pension expansions on memory decline and dementia probability. This analysis extends the current evidence base with a natural experiment design, longer term longitudinal follow-up for cognitive outcomes (7 years), and representation of a sub-Saharan African setting.

## Materials and methods

### Study setting and population

The setting for this study is the Agincourt sub-district of the Mpumalanga province in northeastern South Africa. The sub-district is covered by the Agincourt Health and Socio-Demographic Surveillance System (HDSS) operated by the Medical Research Council/Wits University Rural Public Health and Health Transitions Research Unit. The HDSS covers 31 villages with over 116,000 inhabitants [43,44] and has monitored household membership and vital statistics for all individuals in the study setting since 1992. While conditions have improved in the region since the end of Apartheid, there are limitations in the availability of basic services, as well as high unemployment rates and high food insecurity [43,44]. Thus, social protection programs such as the Older Person’s Grant (OPG) and other governmental cash transfers provide a significant source of income for many individuals and households in this region [43]. Since the current retirement age in South Africa is 60 years old, the OPG is available to individuals who are 60 years old and above [45].

### Data

This study used longitudinal data from the “Health and Ageing in Africa: A Longitudinal Study of an INDEPTH Community [HAALSI]” cohort [46]. The HAALSI cohort study examines the risk factors associated health conditions, including cognitive function and dementia, in an aging rural South African population. To be eligible for study inclusion, men and women had to be 40 + years old as of July 1, 2014 and permanently residing in the area covered by the Agincourt HDSS for 12 months or more. The eligible individuals were randomly sampled from the underlying population-based HDSS and consent to participate was given by 5059 individuals (2345 men and 2714 women). Trained and supervised fieldworkers conducted the survey in three waves every three years between 2014 and 2021 using computer assisted personal interviews, with ongoing field and data monitoring. The fieldworkers conducted the survey interviews in the local XiTsonga language and, to ensure reliability, the responses were translated from English and back translated [46].

This study used de-identified secondary data which was not considered human subjects research by IU IRB.

### Key measures

#### Exposure variable: Men’s eligibility for expanded older person’s grant.

The South African Older Person’s Grant (OPG) (previously Old Age Pension (OAP)) is a means-tested program that provides a monthly cash transfer of R2090 ($110 USD) [20,47]. South African citizens, permanent residents or refugees living in South Africa with R86,280 or less in annual earnings or <R1,227,6000 in assets are eligible to receive the OPG [48]. It is highly likely that the participants in this study meet the eligibility requirement due to the high poverty, food insecurity and unemployment rates in the HAALSI study setting [20,43].

When South Africa’s Older Person’s Grant expanded to the country’s previously excluded population, women were eligible at age 60 and men were eligible at age 65. However, in 2008, eligibility expanded for men to include those aged 63 years and older. In 2009, the age eligibility for men expanded yet again and included men aged 61 years and older. Finally, in 2010, the age eligibility for men was expanded to include men aged 60 years and older. These pension eligibility expansions occurred over a short amount of time and, in turn, led to cohorts of men who were close in age but with variation in the amount of pension income for which they were eligible. These exogenous expansions provide an opportunity to examine the impact of pension transfers plausibly unrelated to other factors that may affect memory decline and dementia probability.

Using respondents’ birth dates and the pension eligibility expansion dates, we grouped HAALSI men into three cohorts: men who turned 65 before the pension eligibility expansions and, in turn, were never eligible to receive pension at an earlier age (pre-transition cohort), men who reached pension eligibility age during the pension eligibility expansion years and were eligible to receive pension one to four years earlier than the pre-transition cohort (transition cohort), and men who reached pension eligibility age after the pension eligibility expansions and, thus, were eligible to receive their pension five years earlier than the pre-transition group (post-transition cohort). Specifically, men in the HAALSI cohort who were born before 1944 were in the pre-transition cohort and never eligible to receive pension prior to age 65. Those born between 1944 and 1949 were in the transition cohort, as they were eligible to receive a pension one to four years earlier than the pre-transition cohort. Those born in 1950 and the subsequent years were in the post-transition cohort, as they were eligible to receive pension a full five years earlier than the pre-transition cohort.

#### Outcome: Rate of memory change over time.

Episodic memory tests were administered by trained local field staff to participants at each data collection wave of the HAALSI study in 2014/15, 2018/19, and 2021/22. The tests included both immediate and delayed recall of a 10-noun list read out loud by the interviewer. Tests were carefully selected and pilot tested to ensure cultural relevance. Testing was administered through an innovative tablet-based approach. Training, support, and supervision were thorough and repeatedly refreshed to ensure the quality of resulting data. Forward and back-translation into XiTsonga was carried out several times to ensure the meaning of the cognitive test was captured in the local vernacular. The immediate and delayed memory recall tests were adapted from a validated test used widely by the Health and Retirement Studies (HRS) [49]. While the immediate and delayed recall items haven’t been validated directly in the Agincourt research area, the HRS cognitive tests have been implemented across various international and low-resource settings [50,51]. Research has confirmed that despite variations in implementation, the cognitive assessments across the HRS’s International Partner Studies measure similar underlying cognitive constructs [50].

To account for the variation in the administration of the memory test between waves, we used confirmatory factor analysis to construct memory factor scores for each time point and then z-standardized the scores to the Wave 1 (baseline) distribution, which was standardized to have a mean of 0 and standard deviation of 1. Thus, the estimates for change in memory over time are for change relative to the baseline distribution. This method of using confirmatory factor analysis to construct factor scores using cognitive measures has been validated by other studies [52–55].

#### Outcome: Dementia probability scores.

Dementia probability scores were estimated for HAALSI participants in Wave 3. To generate dementia probability scores, a consensus-based diagnosis was obtained from the HAALSI Dementia sub-cohort (n = 635) as a “gold standard” [56]. We then used measures available on all HAALSI participants in wave 2 to predict clinical diagnoses in subsequent HAALSI dementia (HAALSI-HCAP) waves. The models included cognitive measures such as immediate and delayed recall, self-rated memory, verbal fluency, and the sum score of days of the week forward and backward, in addition to limitations to activities of daily living and instrumental activities of daily living. We used logistic regression models for the prediction models, with the outcome being the dementia diagnosis based on clinical consensus [57]. This method follows the approach used in the HRS-ADAMS study [58], and allowed us to develop a predictive model within the HAALSI Dementia cohort. We then applied the coefficients from these models to the entire population to obtain dementia probability scores for the full HAALSI sample at Wave 3.

#### Covariates.

We used the following variables to contextualize the study population and create predictive models for the two cognitive outcomes: age (continuous, in years), marital status (never married, separated/divorced, widowed, currently married), level of education (no formal education, primary education, secondary education or higher), employment status (binary for currently employed or not), self-reported literacy (binary for able to read and/or write or not able to read and write), the number of children (discrete), country of origin (born in South Africa; born outside of South Africa), a binary indicator variable representing whether any household member was eligible to receive the Child Support Grant, household-specific random intercepts and a wealth asset index (in 5 quintiles). The wealth asset index variable was created from a principal components analysis on household characteristics and ownership of household items [59].

### Statistical analysis

**Fig 1:**
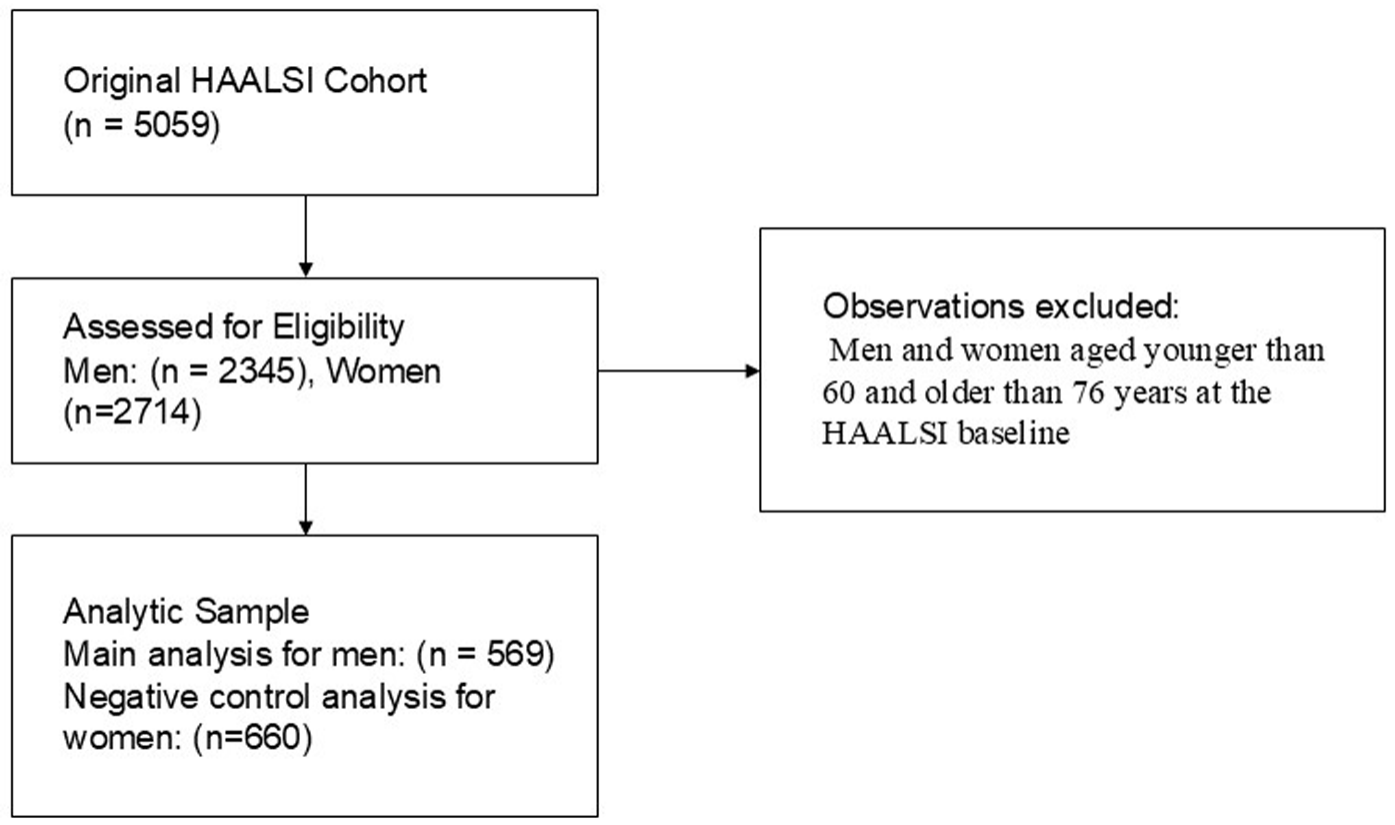
Sample inclusion flow chart.

We used a multi-step modeling approach to isolate the effect of the pension expansion exposure on memory decline and dementia probability outcomes among men. Our analytic goal was to compare observed values of the cognitive outcomes to what would be expected in the absence of the pension expansion, given the sociodemographic profile alone. Fig 1 displays a sample selection flow chart outlining our methods for selecting the study’s analytic sample. First, we age-restricted the sample to only include men aged 60–76 years at the HAALSI baseline to exclude individuals who were aging into pension eligibility over the course of HAALSI follow- up. This also allowed us to reduce sociodemographic differences across the three cohorts and to increase the probability that our estimated effects are related primarily to the exogenous differences in pension eligibility. The restricted cohort allowed us to reasonably draw conclusions for groups of individuals within a similar age range, while keeping a large enough sample size to maintain statistical power. For our primary analysis, we restricted the sample to include only participants with test scores from all three waves of data to capture cognitive decline across the longest time range possible. To account for mortality and attrition in this sample, all final step models were weighted by the inverse probability of mortality and attrition.

For memory decline models, we extracted the person-specific memory slope from Wave 1 to Wave 3 (2014–2021) for all participants in the restricted sample using a linear mixed-effects model. We then fit a linear model to predict the individual memory slopes by using data from participants who were not exposed to any expansions in pension eligibility (pre-transition cohort). This model contained a robust set of variables including age, marital status, level of education, employment status, self-reported literacy, number of children, country of origin, household Child Support Grant eligibility and wealth asset index. We used the coefficients from the model predicting memory slopes from the individuals in the pre-transition cohort to obtain predicted values for the memory slopes in the transition and post-transition cohorts. We then calculated the difference in scores for each person in the transition and post-transition cohorts by subtracting the predicted values derived from the restricted sample linear regression from the observed memory slope values from transition and post-transition cohorts. Finally, we estimated a linear model on the difference score to obtain estimated mean difference scores for each cohort. The model also contained a robust set of variables including age, marital status, level of education, employment status, self-reported literacy, number of children, country of origin, household Child Support Grant eligibility and wealth asset index, to adjust for factors that may impact cognitive function, other than pension eligibility. Practice effects for memory scores were observed between Waves 1 and 2 due to repeated cognitive assessments. Practice effects are expected to be large after the first testing occasion but are less of an issue after the participant is already familiar with the testing procedures (W2 onward). We accounted for this by including a binary indicator variable representing observations from Wave 1 (63)To account for multiple participants in the same household, we incorporated household-specific random intercepts into the models by including a unique identifier for the household that nests individuals within households. Since the missingness for any variable in our model did not exceed 2.5%, we used a complete case following the general rule that if missingness is < 5% then a complete case analysis is sufficient [60].

As a negative control analysis, we recreated the analysis outlined in the above steps in HAALSI women. Since women in South Africa have always (post-1994) been eligible to receive the pension at age 60, they were not exposed to any extra pension eligibility from the South African Older Person’s Grant expansions. Using women as a negative control group serves to check the robustness of our findings and the presence of residual confounding. As women experienced no exogenous variation in pension receipt from eligibility expansions, we would anticipate no effect of pension on our cognitive outcomes if our variables and models are correctly specified [61]. The presence of an effect in both men and women would indicate the presence of residual confounding and that we failed to fully isolate the effect of pension expansion. Additionally, to further check the robustness of our findings, we performed these steps using participants with only two waves of data. This allowed us to test whether our results were sensitive to excluding those who died between Wave 2 and Wave 3.

For the models using dementia probability as the dependent variable, we used a model similar to that outlined above, with a few modifications. First, we restricted the sample to only Wave 3 HAALSI participants. We did this to ensure there was sufficient lag time between the pension expansion eligibility exposure and the outcome. We then fit a linear equation to predict dementia probability by using the data from the participants in the pre-transition cohort. The linear regression was performed to get a linear function to estimate predicted dementia probability for the participants in the transition and post-transition cohorts. We then calculated difference scores for each person in the transition and post-transition cohorts by subtracting the predicted values derived from the restricted sample linear regression from the observed dementia probability values from transition and post-transition cohorts. Finally, we estimated a linear model on the difference score to get estimated mean difference scores for each cohort. The model contains a robust set of variables, including age, marital status, level of education, employment status, self-reported literacy, number of children, country of origin, household Child Support Grant eligibility and wealth asset index, to adjust for potential confounding factors. As with the memory decline outcomes, we also performed a negative control analysis for dementia probability by recreating the analysis outlined in the above steps in HAALSI women. We used both Stata (version 16.1) and R (version 4.1.1) statistical software for all analyses.

## Results

We identified 569 male and 660 female HAALSI participants from the original Wave 1 population who met our study inclusion criteria (Table 1; Fig 1). Across the combined study population, 28% of participants were in the pre-transition cohort, 33% were in the transition cohort, and 39% were in the post-transition cohort. Participants were, on average, 67 years old (SD: 5.1). Participants in the pre-transition cohort were more likely to have no education, more likely to be unemployed, and less likely to be literate compared to those in the transition and post-transition cohorts. The differences in memory decline and dementia probability for individuals in the transition and post-transition cohorts were calculated by subtracting the predicted values (from the restricted sample linear regression) from their observed memory slope and dementia probability values, respectively.

**Table 1:** Sociodemographic characteristics of men in HAALSI cohort, by eligibility for the Older Person’s Grant expansion.

Characteristic	Overall	Pre-Transition	Transition	Post-Transition	p-value^2^
	N = 1,229^1^	N = 344^1^	N = 399^1^	N = 486^1^	
Age	67.2 [5.1]	73.4 [2.9]	67.6 [2.6]	62.5 [1.9]	**<0.001**
Number of Children	5.2 [2.8]	5.0 [3.0]	5.4 [2.9]	5.2 [2.7]	0.06
Male	569 [46%]	237 [49%]	155 [45%]	177 [44%]	0.4
Education					**0.001**
No formal	619 [51%]	204 [60%]	196 [49%]	219 [45%]	
Primary	503 [41%]	117 [34%]	163 [41%]	223 [46%]	
Secondary or more	103 [8.4%]	21 [6.1%]	39 [9.8%]	43 [8.9%]	
*Missing*	4	2	1	1	
Born in South Africa	885 [72%]	242 [70%]	285 [71%]	358 [74%]	0.5
Wealth Index					0.2
Quintile 1	213 [17%]	70 [20%]	66 [17%]	77 [16%]	
Quintile 2	217 [18%]	65 [19%]	63 [16%]	89 [18%]	
Quintile 3	236 [19%]	61 [18%]	78 [20%]	97 [20%]	
Quintile 4	288 [23%]	64 [19%]	98 [25%]	126 [26%]	
Quintile 5	275 [22%]	84 [24%]	94 [24%]	97 [20%]	
Marital Status					**<0.001**
Never married	23 [1.9%]	4 [1.2%]	2 [0.5%]	17 [3.5%]	
Separated/divorced	128 [10%]	28 [8.1%]	49 [12%]	51 [10%]	
Widowed	413 [34%]	148 [43%]	124 [31%]	141 [29%]	
Currently married	665 [54%]	164 [48%]	224 [56%]	277 [57%]	
Employed	78 [6.4%]	11 [3.2%]	20 [5.0%]	47 [9.7%]	**<0.001**
*Missing*	2	1	0	1	
Literate	692 [56%]	163 [47%]	223 [56%]	306 [63%]	**<0.001**
Memory Score [Wave 1]	−0.06 [0.86]	−0.21 [0.87]	0.00 [0.83]	0.00 [0.87]	**<0.001**
Memory Score [Wave 2]	0.26 [0.85]	0.05 [0.92]	0.29 [0.83]	0.39 [0.80]	**<0.001**
Memory Score [Wave 3]	0.20 [0.70]	−0.04 [0.73]	0.23 [0.64]	0.35 [0.70]	**<0.001**
Dementia Probability [Wave 3]	0.12 [0.12]	0.17 [0.14]	0.11 [0.09]	0.10 [0.10]	**<0.001**
*Missing*	34	8	14	12	

### South Africa’s older Person’s grant and longitudinal memory scores

**Fig 2:**
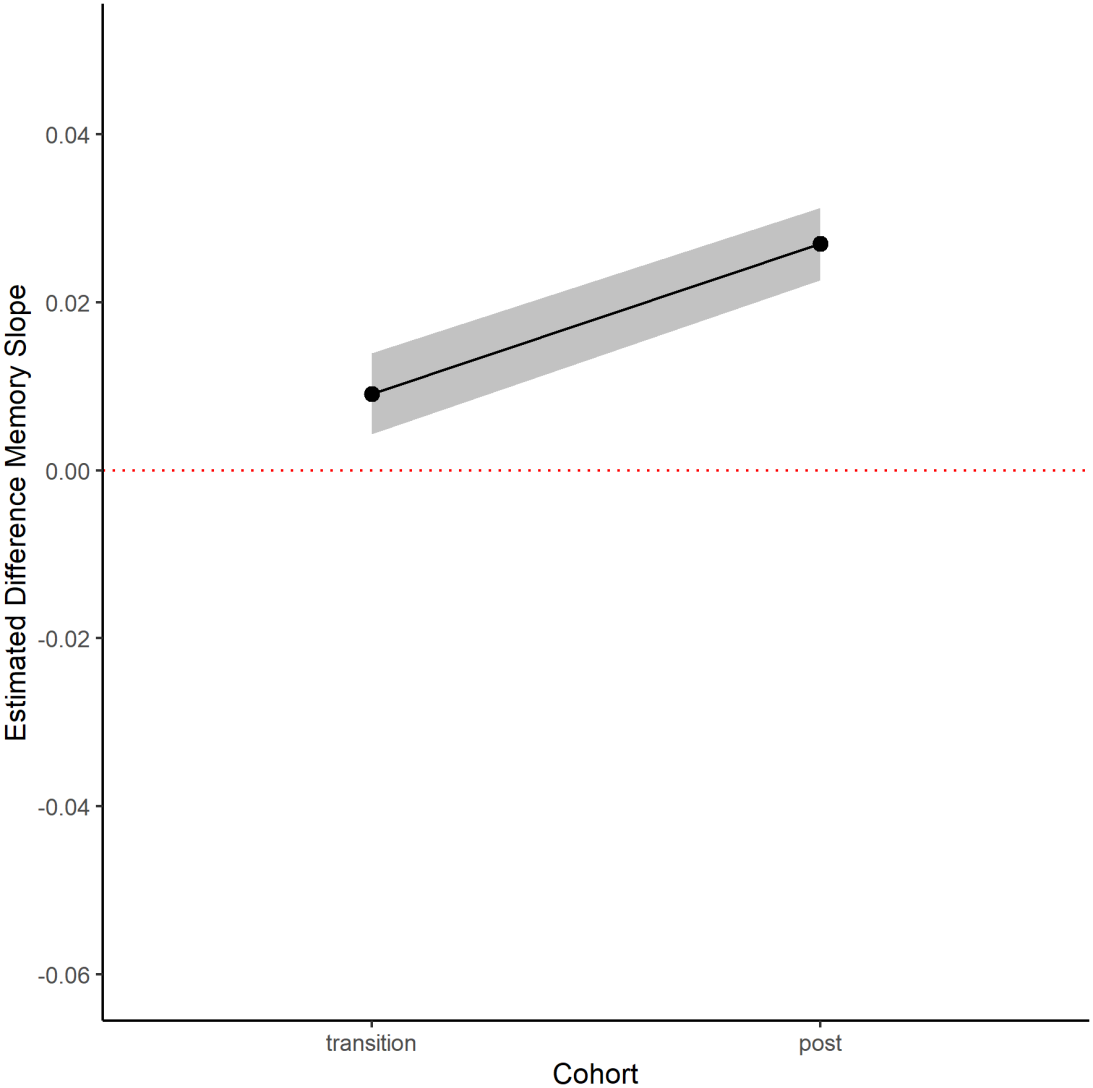
Difference between predicted and observed memory slopes in cohorts of HAALSI men exposed to additional years of pension eligibility (3 waves).

Men in the transition and post-transition cohorts experienced slower memory decline than expected, based on the slope observed in the pre-transition cohort after adjusting for other potential factors that might affect cognition function (see Table 2, Fig 2). In the main analysis, participants in the post-transition cohort were estimated to have memory decline slopes that were less steep than their predicted values by 0.027 SD units per year [95% CI: 0.023, 0.031] between the years 2014 and 2021. Participants in the transition cohort were estimated to have memory decline slopes that were less steep than their predicted values by 0.009 SD units per year [95% CI: 0.004, 0.013]. These results remained robust after the sample size was broadened to include men with only two waves of data (Table 2).

**Table 2:** Difference between predicted and observed memory slopes in cohorts of HAALSI men exposed to additional years of pension eligibility.

	Cohort	Predicted Memory Slope*	Observed Memory Slope	Estimated Difference in SD [95% CI]	P-value
**3 waves**	**Transition**	−0.037	−0.028	0.009 [0.004, 0.013]	0.000
	**Post-Transition**	−0.048	−0.021	0.027 [0.023, 0.031]	0.000
**2 waves**	**Transition**	−0.016	−0.012	0.004 [0.000, 0.007]	0.04
	**Post-Transition**	−0.016	−0.012	0.004 [0.000, 0.006]	0.14

*Transition Cohort: Eligible to receive pension 1–4 years earlier; Post-Transition Cohort: Eligible to receive pension 5 years earlier. Results based on linear regression model with the following predictors: birth year, wealth index, marital status, HIV status, level of education, employment status, literacy level, number of children, country of origin, a binary indicator variable representing whether any household member was eligible for the Child Support Grant and household-specific random intercepts. The models also include a binary indicator variable representing observations from Wave 1 to account for practice effects.

**Fig 3:**
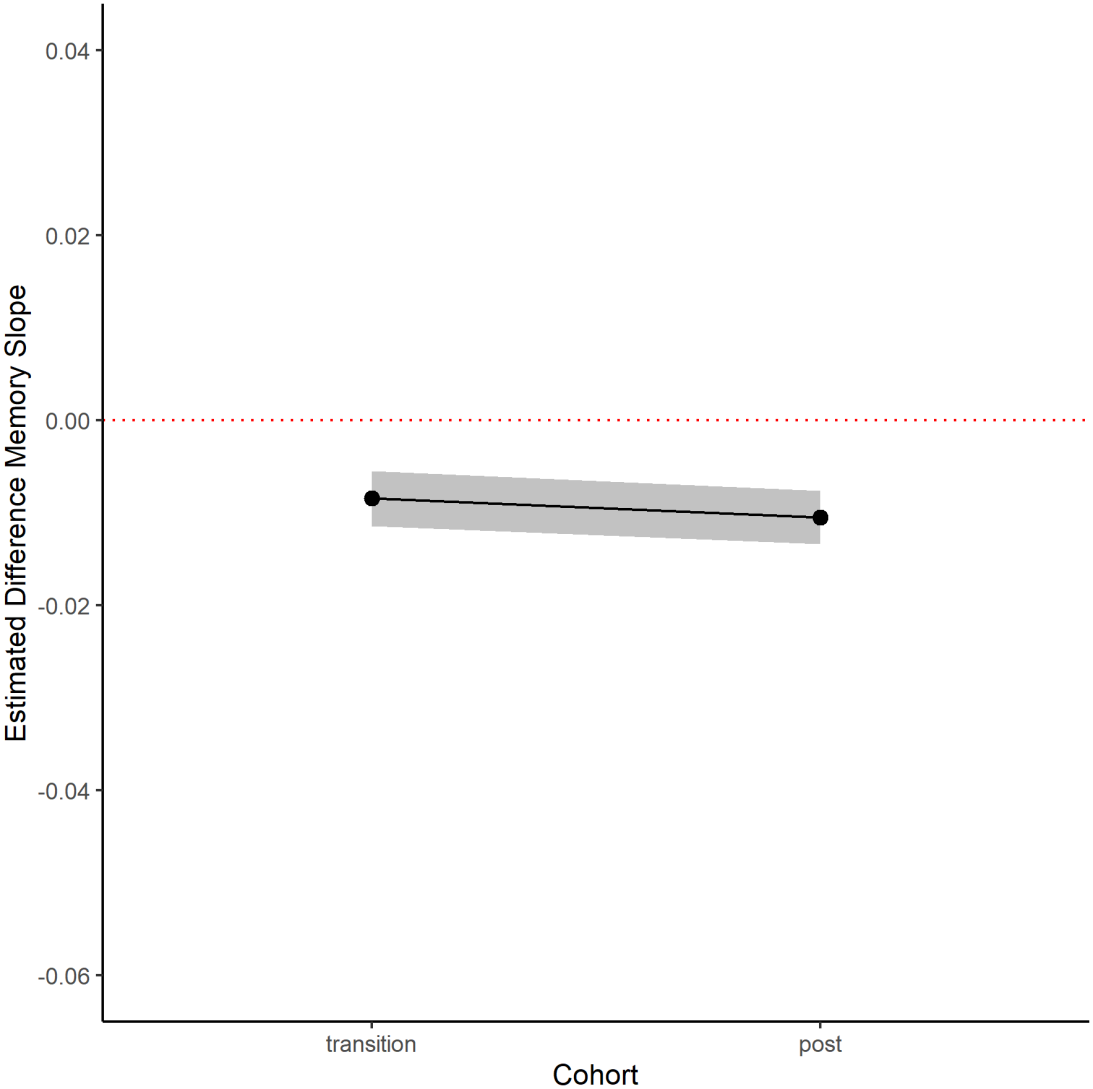
Robustness check: Difference between predicted and observed memory slopes in cohorts of HAALSI women (3 waves).

The observed memory decline slopes for women of the same age as men in the transition cohorts were steeper than their predicted memory decline slopes (Table 3, Fig 3). In the restricted sample of participants with three waves of data (Table 3, Fig 3), women with birth dates in the same range as men in the post-transition cohort had observed memory decline slopes that were 0.011 SD units per year steeper compared to their predicted slopes [95% CI: −0.013, −0.007]. Moreover, women with birth dates in the same range as men in the transition cohort had observed memory decline slopes that were 0.009 SD units per year steeper compared to their predicted slopes [95% CI: −0.011, −0.005]. When the sample was broadened to include women with only two waves of data, the coefficients remained negative, however the magnitude and significance decreased (Table 3).

**Table 3:** Robustness check: Difference between predicted and observed memory slopes in cohorts of HAALSI women.

	Cohort	Predicted Memory Slope*	Observed Memory Slope	Estimated Difference in SD [95% CI]	P-value
**3 waves**	**Transition**	−0.010	−0.019	−0.009 [−0.011, −0.005]	0.000
	**Post-Transition**	−0.008	−0.019	−0.011 [−0.013, −0.007]	0.000
**2 waves**	**Transition**	−0.015	−0.016	−0.001 [−0.004, 0.001]	0.310
	**Post-Transition**	−0.013	−0.016	−0.003 [−0.005, −0.000]	0.019

*Transition Cohort: Eligible to receive pension 1–4 years earlier; Post-Transition Cohort: Eligible to receive pension 5 years earlier. Results based on linear regression model with the following predictors: birth year, wealth index, marital status, HIV status, level of education, employment status, literacy level, number of children, country of origin, a binary indicator variable representing whether any household member was eligible for the Child Support Grant and household-specific random intercepts. The models also include a binary indicator variable representing observations from Wave 1 to account for practice effects.

### South Africa’s Older Person’s Grant and Dementia Probability Scores

**Fig 4:**
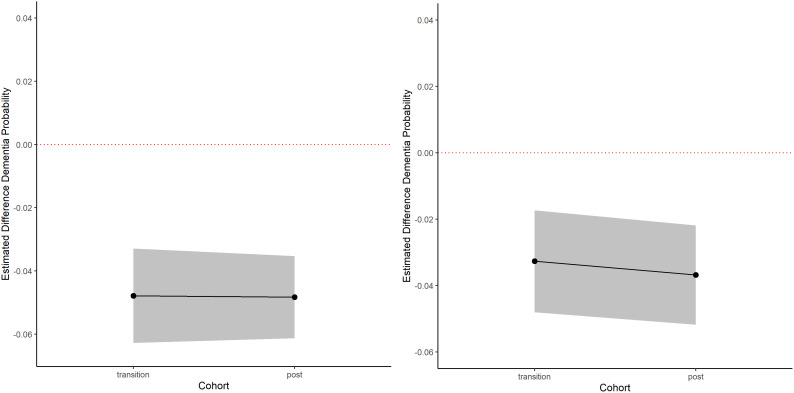
Difference between predicted and observed dementia probability scores in cohorts of HAALSI men exposed to additional years of pension eligibility and negative control for women.

Exposure to expanded pension eligibility was associated with lower dementia probability scores among men in the transition and post-transition cohorts at HAALSI Wave 3 compared to the pre-transition cohort (Table 4, Fig 4). Men in the post-transition cohort had observed dementia probability scores that were 5.2 percentage points lower than their predicted scores [95% CI: −0.062, −0.032]. Moreover, the transition cohort had observed dementia probability scores that were 4.8 percentage points lower than their predicted scores [95% CI: −0.062, −0.032]. In the negative control analysis, women with birth dates in the same range as men in the post-transition cohort had observed dementia probability scores that were 4.3 percentage points lower than their predicted scores [95% CI: −0.051, −0.021]. Women with the same birth dates as men in the transition cohort had dementia probability scores that were 3.6 percentage points lower than their predicted scores [95% CI: −0.048, −0.017].

**Table 4:** Difference between predicted and observed dementia probability scores.

	Cohort	Predicted Dementia Probability Score*	Observed Dementia Probability Score	Estimated Difference in SD [95% CI]	P-value
**Men**	**Transition**	0.141	0.093	−0.048 [−0.062, −0.032]	0.000
	**Post-Transition**	0.151	0.099	−0.052 [−0.062, −0.032]	0.000
**Women**	**Transition**	0.164	0.128	−0.036 [−0.048, −0.017]	0.000
	**Post-Transition**	0.150	0.107	−0.043 [−0.051, −0.021]	0.000

*Transition Cohort: Eligible to receive pension 1–4 years earlier; Post-Transition Cohort: Eligible to receive pension 5 years earlier. Results based on linear regression model with the following predictors: birth year, wealth index, marital status, HIV status, level of education, employment status, literacy level, number of children, country of origin, a binary indicator variable representing whether any household member was eligible for the Child Support Grant and household-specific random intercepts. The models also include a binary indicator variable representing observations from Wave 1 to account for practice effects.

## Discussion

This study is among the first to employ a quasi-experimental method to estimate the impact of pension programs on long-term memory decline and dementia probability. We did this by exploiting exogenous expansions in eligibility for the Older Person’s Grant for men in South Africa that occurred between the years 2008 and 2010. These expansions resulted in some men receiving their pension one to five years earlier than original pension eligibility guidelines depending on their birth date at the time of the pension expansions. We used two cognitive outcomes to explore these associations: episodic memory decline and dementia probability scores. We found consistent evidence that earlier pension eligibility was associated with slower memory decline and lower dementia probability in men aged 60–70 in rural South Africa. Additionally, there was evidence of a dose-response relationship where increasing years of additional pension eligibility resulted in stronger protective effects on memory decline. We did not observe this same protective relationship with memory decline in our negative control analysis for women who were not exposed to any earlier pension eligibility. Thus, our findings suggest that expanded pension eligibility resulted in cognitive health protections for older men in this setting. Moreover, Agincourt is a rapidly transitioning rural South African environment due to the fast-evolving health, population and social transitions underway. Such transitions characterize much of rural South Africa and the region. In turn, our results may be generalizable to other rapidly transitioning rural South African settings.

Overall, our findings are in alignment with the small body of literature that has examined the relationship between pension receipt and subsequent cognitive function in older adults. Research across multiple countries has demonstrated a protective effect of pension receipt on cognitive health [8,9,17,20,38,41], with several studies demonstrating a clinically meaningful improvement in cognitive function [8,9,38]. Previous longitudinal research across Korea, China, and Mexico found that increasing levels of pension receipt contributed to higher levels of cognitive function. In Mexico and Korea, specifically, pension receipt did not just delay decline but produced improvements in word recall and cognitive function tests, respectively [8,41]. In contrast, while we found that earlier pension receipt slowed memory decline relative to the comparison group, it did not result in overall improvements in memory scores. This differences are likely related to the longer time horizon and time between observations during our study compared to the pre-existing literature. Our study contributes a novel approach to this literature by using longitudinal data on episodic memory scores to measure cognitive decline. Moreover, we triangulated findings with analyses of pension eligibility impacts on dementia probability scores.

Prior research has shown socioeconomic status and income to be important predictors of cognitive function and decline [6,8–13,15–18]. Higher income individuals typically have access to better quality education and health care, access to better nutrition, healthier behaviors, and better living conditions. Moreover, an increase in income may impact memory decline and ADRD risk by building cognitive reserve. Cognitive reserve improves the brain’s ability to cope with brain changes and damage that occurs in aging [62]. Increased income can increase an individual’s access to cognitively stimulating exposures, such as intellectual and social activities, that can build cognitive reserve and slow cognitive decline from aging [36,58]. Moreover, research indicates that stress is associated with cognitive decline [63,64]. Cash transfers for low-income older adults who may experience financial stress may be an impactful intervention for building cognitive reserve and slowing cognitive decline. It is important to note that there are other health and population transitions that are occurring in South Africa that likely impact cognitive function and the rate of cognitive decline for the older population. One notable transition is that life expectancies have increased due to improved health and economic conditions in the last several decades. In turn, the country is experiencing an epidemiological transition to more chronic diseases over infectious diseases and the rates of Alzheimer’s Disease and Related Dementias are predicted to increase significantly [20].

For men, the observed slope of memory decline was significantly more gradual compared to the predicted slope, representing a slower decline in memory function. This effect increased as the number of years of additional pension increased. In contrast, we observed steeper memory decline in the observed compared to predicted slopes among women in our negative control analysis. The magnitude of effect was consistent across expansion cohorts for women. Since the effect was similar across expansion cohorts for women but not men, this likely represents a systematic underestimation of memory scores by our predictive model rather than residual confounding. Therefore, the magnitude of our results could be biased but we anticipate that the relative effect that we detected among men would be accurate. That said, we also found that men and women in the same expansion cohorts both had lower observed probabilities of dementia compared with their predicted probabilities. While the dementia probability scores for women were smaller in magnitude than for men [Transition Cohort: −3.6pp vs −4.8pp; Post-Transition Cohort: −4.3pp vs −5.2pp], a statistical comparison was not carried out to confirm the difference due to the difference in samples. Still, it is important to consider why we may have found differential results by gender for episodic memory scores but not for dementia probability. This could indicate that sources of residual confounding for dementia probability were not present for memory scores. As dementia probability is a more comprehensive measure of cognitive function, it is likely that it could be impacted by a larger range of factors we were unable to account for compared to memory scores. However, in this study context, individuals share their pension income with others in their household [62,64]. Therefore, there could also be spillover effects from the South African pension expansions if the men shared the money with women in their household. Further research is needed to examine the potential spillover effects of cash transfers for other older adults in the household and the impacts this has on cognitive decline and ADRD risk.

Our results should be interpreted within the limitations of the restrictions we made on the sample composition. We restricted the sample to ages 60–76 to reduce sociodemographic differences across the three cohorts which reduced our sample size. Moreover, we were only able to analyze the impacts of pension eligibility expansions for men, since women were not exposed to extra pension eligibility. While we hypothesized that there may have been positive spillover effects for women living in the pension eligibility expansion cohort households, we were not able to measure these effects due to limitations in the natural experiment. Moreover, our study area was restricted to one rural South African setting. While this setting is representative of rapidly transitioning contexts across rural South Africa, cash transfers may have different impacts for those living in other geographic settings, and in more urban settings.

## Conclusion

In settings where social environments are improving and medical advancements are more accessible, life expectancies are increasing while the burden of ADRD is growing around the world. To address this growing burden, it is critical for policymakers to implement evidence-based policies that support healthy brain ageing and reduce ADRD risk. This preventative approach is particularly important in LMICs, which often lack adequate formal health and social care for individuals with dementia. Diverse social and cultural factors influence whether persons with ADRD remain at home and connected to communities and social networks, rather than admitted (as in a western model) to ‘homes for the aged’ and related facilities. Thus, for South Africans with ADRD living at home with family, old age pensions may be a crucial financial safety net for their households. This study provides evidence that cash transfers in the form of public pensions may slow memory decline and lower the probability of dementia. Moreover, the results suggest that cash transfers to older adults in the form of pensions may have spillover effects for other adults in the household. These findings provide evidence that cash transfers, such as pensions or other income transfers for older adults, are promising policy interventions for reducing ADRD risk and promoting health ageing.

## Supporting information

S1 File Difference between predicted and observed memory slopes in cohorts of HAALSI men exposed to additional years of pension eligibility (2 waves). (DOCX)

S2 File Robustness check: Difference between predicted and observed memory slopes in cohorts of HAALSI women (2 waves). (DOCX)

## References

[1] Nichols E, Feigin VL, Collaborators GDF. Estimation of the Global Prevalence of Dementia in 2019 and Forecasted Prevalence in 2050: An Analysis for the Global Burden of Disease Study 2019. Lancet Public Health. 2022 [cited 2022 Mar 6]. Available from: https://openrepository.aut.ac.nz/handle/10292/14823 10.1016/S2468-2667(21)00249-8 PMC8810394 34998485

[2] Javaid SF, Giebel C, Khan MA, Hashim MJ. Epidemiology of Alzheimer’s disease and other dementias: rising global burden and forecasted trends [Internet]. F1000Research; 2021 [cited 2022 Jan 10]. Available from: https://f1000research.com/articles/10-425

[3] 2021 Alzheimer’s disease facts and figures. Alzheimers Dement. 2021;17(3):327–406. 33756057 10.1002/alz.12328

[4] Clouston SAP, Smith DM, Mukherjee S, Zhang Y, Hou W, Link BG, et al. Education and Cognitive Decline: An Integrative Analysis of Global Longitudinal Studies of Cognitive Aging. J Gerontol B Psychol Sci Soc Sci. 2020;75(7):e151–60. doi: 10.1093/geronb/gbz053 31059564 PMC7424268

[5] Hughes ML, Agrigoroaei S, Jeon M, Bruzzese M, Lachman ME. Change in Cognitive Performance From Midlife Into Old Age: Findings from the Midlife in the United States (MIDUS) Study. J Int Neuropsychol Soc. 2018;24(8):805–20. doi: 10.1017/S1355617718000425 30019663 PMC6170692

[6] Insel PS, Mattsson N, Mackin RS, Schöll M, Nosheny RL, Tosun D, et al. Accelerating rates of cognitive decline and imaging markers associated with β-amyloid pathology. Neurology. 2016;86(20):1887–96. 27164667 10.1212/WNL.0000000000002683 PMC4873684

[7] Thorvaldsson V, Hofer SM, Berg S, Skoog I, Sacuiu S, Johansson B. Onset of terminal decline in cognitive abilities in individuals without dementia. Neurology. 2008;71(12):882–7. 18753475 10.1212/01.wnl.0000312379.02302.ba

[8] Aguila E, Kapteyn A, Smith JP. Effects of income supplementation on health of the poor elderly: The case of Mexico. Proc Natl Acad Sci. 2015;112(1). 10.1073/pnas.1414453112 PMC4291674 25535388

[9] Ayyagari P, Frisvold D. The impact of social security income on cognitive function at older ages. Am J Health Econ. 2016;2(4):463–88.

[10] Clarke PJ, Weuve J, Barnes L, Evans DA, Mendes de Leon CF. Cognitive decline and the neighborhood environment. Ann Epidemiol. 2015;25(11):849–54. 26253697 10.1016/j.annepidem.2015.07.001 PMC4609590

[11] Danielewicz AL, Wagner KJP, d’Orsi E, Boing AF. Is cognitive decline in the elderly associated with contextual income? Results of a population-based study in southern Brazil. Cad Saúde Pública. 2016;32:e00112715. 10.1590/0102-311X00112715 27192028

[12] Koster A, Penninx BWJH, Bosma H, Kempen GIJM, Newman AB, Rubin SM, et al. Socioeconomic differences in cognitive decline and the role of biomedical factors. Ann Epidemiol. 2005;15(8):564–71. doi: 10.1016/j.annepidem.2005.02.008 15922627

[13] Luo Y, Zhang L, Pan X. Neighborhood environments and cognitive decline among middle-aged and older people in China. J Gerontol Ser B. 2019;74(7):e60–71. 10.1093/geronb/gbz016 30726959

[14] McLean J, Krishnadas R, Batty GD, Burns H, Deans KA, Ford I. Early life socioeconomic status, chronic physiological stress and hippocampal N-acetyl aspartate concentrations. Behav Brain Res. 2012;235(2):225–30. 22917526 10.1016/j.bbr.2012.08.013

[15] Nikolov P, Hossain MS. Do pension benefits accelerate cognitive decline in late adulthood? Evidence from rural China. J Econ Behav Organ. 2023;205:594–617.

[16] Okamoto S. Socioeconomic factors and the risk of cognitive decline among the elderly population in Japan. Int J Geriatr Psychiatry. 2019;34(2):265–71. doi: 10.1002/gps.5015 30370551

[17] Peng C, Burr JA, Han SH. Cognitive function and cognitive decline among older rural Chinese adults: the roles of social support, pension benefits, and medical insurance. Aging Mental Health. 2023;27(4):771–9. 35702759 10.1080/13607863.2022.2088693 PMC10460523

[18] Yu X, Zhang W, Kobayashi LC. Duration of poverty and subsequent cognitive function and decline among older adults in China, 2005-2018. Neurology. 2021;97(7):e739–46. 10.1212/WNL.0000000000012343 PMC8377873 34099525

[19] Rosenberg M, Beidelman E, Chen X, Canning D, Kobayashi L, Kahn K, et al. The impact of a randomized cash transfer intervention on mortality of adult household members in rural South Africa, 2011-2022. Soc Sci Med. 2023;324:115883. 37023659 10.1016/j.socscimed.2023.115883 PMC10124166

[20] Jock J, Kobayashi L, Chakraborty R, Chen X, Wing C, Berkman L, et al. Effects of pension eligibility expansion on men’s cognitive function: findings from rural South Africa. J Aging Soc Policy. 2023;1–20. 10.1080/08959420.2023.2195785 PMC10533724 36975023

[21] Chakraborty R, Kobayashi LC, Jock J, Wing C, Chen X, Phillips M, et al. Child Support Grant expansion and cognitive function among women in rural South Africa: findings from a natural experiment in HAALSI cohort. MedRxiv Prepr Serv Health Sci. 2023. doi: 2023.02.18.23286130 10.1371/journal.pone.0297673 PMC10917272 38446751

[22] Riumallo Herl C, Kabudula C, Kahn K, Tollman S, Canning D. Pension exposure and health: Evidence from a longitudinal study in South Africa. J Econ Ageing. 2022;23:None. doi: 10.1016/j.jeoa.2022.100411 36505964 PMC9731801

[23] Aguila E, Kapteyn A, Smith JP. Effects of income supplementation on health of the poor elderly: the case of Mexico. Proc Natl Acad Sci U S A. 2015;112(1). 10.1073/pnas.1414453112 PMC4291674 25535388

[24] Rosalini MHP, Probst LF, Cunha IP da, Gondinho BVC, Cortellazzi KL, Possobon R de F, et al. Quality of life, cohesion and adaptability in beneficiary families of the “Bolsa Família” Program. Cien Saude Colet. 2019;24(1):307–14. doi: 10.1590/1413-81232018241.30592016 30698263

[25] Soares FV, Ribas RP, Osório RG. Evaluating the Impact of Brazil’s Bolsa Família: Cash Transfer Programs in Comparative Perspective. Lat Am Res Rev. 2010;45(2):173–90.

[26] Rivera JA, Sotres-Alvarez D, Habicht JP, Shamah T, Villalpando S. Impact of the Mexican program for education, health, and nutrition [Progresa] on rates of growth and anemia in infants and young children: a randomized effectiveness study. JAMA. 2004;291(21):2563–70. 15173147 10.1001/jama.291.21.2563

[27] Herd P, Schoeni RF, House JS. Upstream solutions: does the supplemental security income program reduce disability in the elderly? Milbank Q. 2008;86(1):5–45. doi: 10.1111/j.1468-0009.2007.00512.x 18307476 PMC2690339

[28] Arno PS, House JS, Viola D, Schechter C. Social security and mortality: the role of income support policies and population health in the United States. J Public Health Policy. 2011;32(2):234–50. doi: 10.1057/jphp.2011.2 21326333 PMC3148579

[29] Haushofer J, Shapiro J. The short-term impact of unconditional cash transfers to the poor: experimental evidence from Kenya. Q J Econ. 2016;131(4):1973–2042. doi: 10.1093/qje/qjw025 33087990 PMC7575201

[30] de Walque D, Fernald L, Gertler P, Hidrobo M. Cash Transfers and Child and Adolescent Development. In: Bundy DAP, de Silva N, Horton S, Jamison DT, Patton GC, editors. Child and Adolescent Health and Development. 3rd edition. Washington [DC]: The International Bank for Reconstruction and Development/ The World Bank. 2017. 30212143

[31] J Carter D, Daniel R, Torrens AW, N Sanchez M, Maciel ELN, Bartholomay P, et al. The impact of a cash transfer programme on tuberculosis treatment success rate: a quasi-experimental study in Brazil. BMJ Glob Health. 2019;4(1):e001029. doi: 10.1136/bmjgh-2018-001029 30740248 PMC6347926

[32] Lee S, Ku I, Shon B. The Effects of Old-Age Public Transfer on the Well-Being of Older Adults: The Case of Social Pension in South Korea. J Gerontol B Psychol Sci Soc Sci. 2019;74(3):506–15. doi: 10.1093/geronb/gbx104 28977644 PMC6377039

[33] Riumallo-Herl C, Aguila E. The effect of old-age pensions on health care utilization patterns and insurance uptake in Mexico. BMJ Glob Health. 2019;4(6):e001771. doi: 10.1136/bmjgh-2019-001771 31798987 PMC6861075

[34] Case A. Does Money Protect Health Status? Evidence from South African Pensions [Internet]. National Bureau of Economic Research; 2001 [cited 2023 Sep 19]. [Working Paper Series]. Available from: https://www.nber.org/papers/w8495

[35] Niño-Zarazúa M, Barrientos A, Hickey S, Hulme D. Social Protection in Sub-Saharan Africa: Getting the Politics Right. World Development. 2012;40(1):163–76.

[36] Stern Y. What is cognitive reserve? Theory and research application of the reserve concept. J Int Neuropsychol Soc. 2002;8(3):448–60. 11939702

[37] Vance DE, Woodley RA. Strengths and distress in adults who are aging with HIV: a pilot study. Psychol Rep. 2005;96(2):383–6. doi: 10.2466/pr0.96.2.383-386 15941113

[38] Cheng L, Liu H, Zhang Y, Zhao Z. The health implications of social pensions: Evidence from China’s new rural pension scheme. J Comp Econ. 2018;46:53–77.

[39] Ayyagari P, Frisvold D. The impact of social security income on cognitive function at older ages full access. Am J Health Econ. 2016;2(4):463–88.

[40] Ayyagari P, Frisvold D. The Impact of Social Security Income on Cognitive Function at Older Ages. Am J Health Econ. 2016 Nov;2(4):463–88.

[41] Hwang I, Lee T-J. Health improvements of older adults based on benefit duration: Lessons from Korean social pension policies. Soc Sci Med. 2022;315:115514. doi: 10.1016/j.socscimed.2022.115514 36395599

[42] Peng C, Burr JA, Han SH. Cognitive function and cognitive decline among older rural Chinese adults: the roles of social support, pension benefits, and medical insurance. Aging Ment Health. 2023;27(4):771–9. doi: 10.1080/13607863.2022.2088693 35702759 PMC10460523

[43] Kahn K, Collinson MA, Gómez-Olivé FX, Mokoena O, Twine R, Mee P, et al. Profile: Agincourt health and socio-demographic surveillance system. Int J Epidemiol. 2012;41(4):988–1001. doi: 10.1093/ije/dys115 22933647 PMC3429877

[44] Collinson MA. Striving against adversity: the dynamics of migration, health and poverty in rural South Africa. Glob Health Action. 2010;3(1):5080. 10.3402/gha.v3i0.5080 PMC2882287 20531981

[45] Farrell MT, Bassil DT, Guo M, Glymour MM, Wagner RG, Tollman S. Estimating dementia prevalence using remote diagnoses and algorithmic modelling: a population-based study of a rural region in South Africa. Lancet Glob Health. 2024;12(12):2003–11. 39577957 10.1016/S2214-109X(24)00325-5 PMC11987161

[46] Gómez-Olivé FX, Montana L, Wagner RG, Kabudula CW, Rohr JK, Kahn K. Cohort profile: Health and ageing in Africa: a longitudinal study of an INDEPTH community in South Africa [HAALSI]. Int J Epidemiol. 2018;47(3):689–90. 29325152 10.1093/ije/dyx247 PMC6005147

[47] Ralston M, Schatz E, Menken J, Gómez-Olivé FX, Tollman S. Who Benefits--Or Does not--From South Africa’s Old Age Pension? Evidence from Characteristics of Rural Pensioners and Non-Pensioners. Int J Environ Res Public Health. 2015;13(1):85. doi: 10.3390/ijerph13010085 26712777 PMC4730476

[48] South African Government. Let’s grow South Africa together [Internet]. [cited 2022 Sep 7]. Available from: https://www.gov.za/

[49] Gómez-Olivé FX, Montana L, Wagner RG, Kabudula CW, Rohr JK, Kahn K, et al. Cohort Profile: Health and Ageing in Africa: A Longitudinal Study of an INDEPTH Community in South Africa (HAALSI). Int J Epidemiol. 2018;47(3):689–690j. doi: 10.1093/ije/dyx247 29325152 PMC6005147

[50] Kobayashi LC, Gross AL, Gibbons LE, Tommet D, Sanders RE, Choi SE, et al. You say tomato, I say radish: can brief cognitive assessments in the US Health Retirement Study be harmonized with its International Partner Studies?. The Journals of Gerontology: Series B. 2020. 10.1093/geronb/gbaa205 PMC8557836 33249448

[51] Weir D, Lay M, Langa K. Economic development and gender inequality in cognition: a comparison of China and India, and of SAGE and the HRS sister studies. J Econ Ageing. 2014;4:114–25. doi: 10.1016/j.jeoa.2014.08.002 25506546 PMC4260415

[52] Wallace J. Confirmatory factor analysis of the cognitive failures questionnaire: evidence for dimensionality and construct validity. Personal Individ Differ. 2004;37(2):307–24.

[53] DeJong J, Donders J. A confirmatory factor analysis of the California Verbal Learning Test--Second Edition (CVLT-II) in a traumatic brain injury sample. Assessment. 2009;16(4):328–36. doi: 10.1177/1073191109336989 19546480

[54] Gross AL, Power MC, Albert MS, Deal JA, Gottesman RF, Griswold M, et al. Application of Latent Variable Methods to the Study of Cognitive Decline When Tests Change over Time. Epidemiology Camb Mass. 2015;26(6):878–87. doi: 10.1097/EDE.0000000000000379 26414855 PMC4819068

[55] Park LQ, Gross AL, McLaren DG, Pa J, Johnson JK, Mitchell M, et al. Confirmatory factor analysis of the ADNI Neuropsychological Battery. Brain Imaging Behav. 2012;6(4):528–39. doi: 10.1007/s11682-012-9190-3 22777078 PMC3538867

[56] Bassil DT, Farrell MT, Weerman A, Guo M, Wagner RG, Brickman AM, et al. Feasibility of an online consensus approach for the diagnosis of cognitive impairment and dementia in rural South Africa. Alzheimers Dement (Amst). 2023;15(2):e12420. doi: 10.1002/dad2.12420 37025188 PMC10072202

[57] Farrell MT, Bassil DT, Guo M, Glymour MM, Langa KM, Wagner RG. Estimating dementia prevalence in rural South Africa with remote consensus diagnoses and algorithmic modeling. Alzheimers Dement. 2023;19(S22):e077769.

[58] Crimmins EM, Kim JK, Langa KM, Weir DR. Assessment of cognition using surveys and neuropsychological assessment: the Health and Retirement Study and the Aging, Demographics, and Memory Study. J Gerontol B Psychol Sci Soc Sci. 2011;66(Suppl 1):i162–71. doi: 10.1093/geronb/gbr048 21743047 PMC3165454

[59] Kabudula CW, Houle B, Collinson MA, Kahn K, Tollman S, Clark S. Assessing changes in household socioeconomic status in rural South Africa, 2001-2013: A distributional analysis using household asset indicators. Soc Indic Res. 2017;133(3):1047–73. 28931968 10.1007/s11205-016-1397-z PMC5579134

[60] Lloyd-Sherlock P, Barrientos A, Moller V, Saboia J. Pensions, poverty and wellbeing in later life: Comparative research from South Africa and Brazil. J Aging Stud. 2012;26(3):243–52.

[61] Lipsitch M, Tchetgen Tchetgen E, Cohen T. Negative controls: a tool for detecting confounding and bias in observational studies. Epidemiology. 2010;21(3):383–8. doi: 10.1097/EDE.0b013e3181d61eeb 20335814 PMC3053408

[62] Stern Y. Cognitive reserve. Neuropsychologia. 2009 Aug 1;47(10):2015–28. 19467352 10.1016/j.neuropsychologia.2009.03.004 PMC2739591

[63] Boals A, Banks JB. Stress and cognitive functioning during a pandemic: Thoughts from stress researchers. Psychol Trauma. 2020;12(S1):S255–7. doi: 10.1037/tra0000716 32463284

[64] Chen Y, Liang Y, Zhang W, Crawford JC, Sakel KL, Dong X. Perceived Stress and Cognitive Decline in Chinese-American Older Adults. J Am Geriatr Soc. 2019;67(S3):S519–24. doi: 10.1111/jgs.15606 31403192

