# Supplementary material for: Effects of pension eligibility expansion on men’s memory decline and dementia probability: Findings from the HAALSI cohort in rural South Africa, 2014–2021
Source: PLoS One. 2025 Jun 25;20(6):e0326321. doi: 10.1371/journal.pone.0326321 (PMC12193015; doi:10.1371/journal.pone.0326321)

**SI: Difference between predicted and observed memory slopes in cohorts of HAALSI men exposed to additional years of pension eligibility (2 waves)**


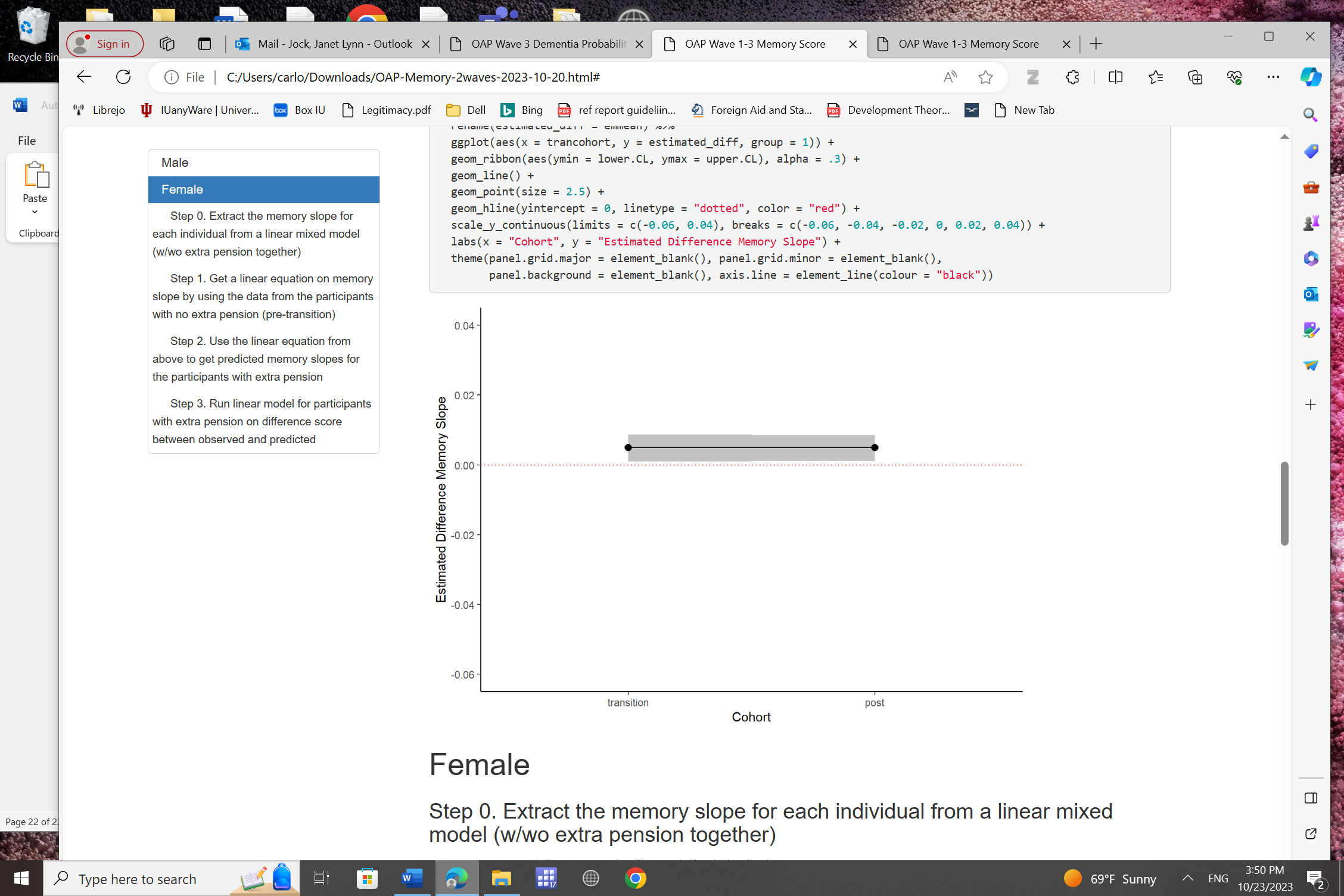

Supplement: S1 File — (DOCX) [file pone.0326321.s001.docx]
